# Permanent Draft Genome Sequence of *Photorhabdus temperata* Strain Hm, an Entomopathogenic Bacterium Isolated from Nematodes

**DOI:** 10.1128/genomeA.00974-17

**Published:** 2017-09-14

**Authors:** Shimaa Ghazal, Erik Swanson, Stephen Simpson, Krystalynne Morris, Feseha Abebe-Akele, W. Kelley Thomas, Kamal M. Khalil, Louis S. Tisa

**Affiliations:** a University of New Hampshire, Durham, New Hampshire, USA; b Applied Microbial Genetics Laboratory, Genetics and Cytology Department, Genetic Engineering & Biotechnology Division, National Research Center, Dokki, Cairo, Egypt

## Abstract

*Photorhabdus temperata* strain Hm is an entomopathogenic bacterium that forms a symbiotic association with *Heterorhabditis* nematodes. Here, we report a 5.0-Mbp draft genome sequence for *P. temperata* strain Hm with a G+C content of 44.1% and containing 4,226 candidate protein-encoding genes.

## GENOME ANNOUNCEMENT

Members of the genus *Photorhabdus* maintain two distinct lifestyles as insect pathogens and in a symbiotic relationship with the entomopathogenic *Heterorhabditid* nematodes (for reviews, see references 1–3). The *Heterorhabditis* nematodes carry a monoculture of *Photorhabdus* within the anterior region of the infective juvenile (IJ) nematode’s intestine (4, 5) and actively seek insect prey in the soil. The nematodes infect a wide range of insect hosts by entering through natural openings or by burrowing directly through the insect cuticle. Once inside the insect, the nematodes regurgitate the bacteria into the hemolymph (4). The bacteria kill the insect within 48 h by releasing highly virulent toxins (6–9). As the bacteria enter the stationary phase of their growth cycle, they secrete extracellular enzymes that aid in breaking down insect tissue, thereby providing nutrients for both the bacteria and the nematodes. The bacteria also generate essential growth factors for nematode growth and development. The growth and development of *Heterorhaabditis* nematodes have an obligate requirement for their specific bacterial symbiont (10). The bacteria also release antibiotics to prevent secondary invaders and putrefaction of the insect carcass (11, 12). After several days of feeding, the nematodes and bacteria reassociate and leave in search of a new insect host.

Members of *Photorhabdus* are classified taxonomically into one of three species, *P. luminescens*, *P. temperata*, or *P. asymbiotica* (13–15). Several subspecies are recognized. Our understanding of these bacteria has been greatly enhanced by genome sequencing of the three established species, including that of *P. luminescens* TT01 (16), *P. asymbiotica* ATCC 43949 (17), *P. temperata* subsp. *khanii* NC19 (18), *P. temperata* Meg1 (19), *P. luminescens* BA1 (20), *P. luminescens* subsp. *laumondii* HP88 (21), *P. asymbiotica* Kingcliff (22), *P. temperata* subsp. *temperata* M121 (23), *P. luminescens* subsp. PB45.5 (24), and *P. asymbiotica* supbsp. australis PB68.1 (24). Here, we present a draft genome sequence for *P. temperata* strain Hm, which was isolated from *Heterorhabditis* nematodes found in Georgia (10).

The draft genome sequence of *P. temperata* strain Hm was generated at the Hubbard Genome Center (University of New Hampshire, Durham, NH) using Illumina technology (25) techniques. A standard Illumina shotgun library was constructed and sequenced using the Illumina HiSeq2500 platform, which generated 15,461,198 reads (150-bp insert size) totaling 2,303.7 Mbp. The Illumina sequence data were trimmed by Trimmomatic version 0.32 (26) and assembled using SPAdes version 3.5 (26) and ALLPaths-LG version r52488 (27). The final draft assembly contained 151 contigs with an *N*_50_ contig size of 71.2 kb and 356.8× coverage of the genome. The final assembled genome contained a total sequence length of 5,003,482 bp with a G+C content of 44.1%. The assembled *P. temperata* strain Hm genome was annotated via the NCBI Prokaryotic Genome Annotation Pipeline (PGAP) and resulted in 4,226 candidate protein-encoding genes and 68 tRNA and 4 rRNA regions.

### Accession number(s).

This whole-genome shotgun project has been deposited at DDBJ/EMBL/GenBank under the accession number MBJU00000000. The version described in this paper is MBJU01000000.
